# Supplementary material for: DNA methylation age acceleration is associated with risk of diabetes complications
Source: Commun Med (Lond). 2023 Feb 10;3:21. doi: 10.1038/s43856-023-00250-8 (PMC9918553; doi:10.1038/s43856-023-00250-8)
Supplement: Supplementary file 2 — Supplementary Material [file 43856_2023_250_MOESM2_ESM.pdf]

DNA methylation age acceleration is associated with risk of diabetes complications

Valentin Max Vetter[1], Johanne Spieker[1], Yasmine Sommerer[2], Nikolaus Buchmann[3], Christian Humberto Kalies[1], Vera Regitz-Zagrosek[4, 5], Lars Bertram[2, 6], Ilja Demuth[1, 7]

[1]Charité – Universitätsmedizin Berlin, corporate member of Freie Universität Berlin and Humboldt-Universität zu Berlin, Department of Endocrinology and Metabolic Diseases (including Division of Lipid Metabolism), Biology of Aging working group, Augustenburger Platz 1, 13353 Berlin, Germany

[2]Lübeck Interdisciplinary Platform for Genome Analytics (LIGA), University of Lübeck, Lübeck, Germany

[3]Department of Cardiology, Charité – University Medicine Berlin Campus Benjamin Franklin, Berlin, Germany

[4]Institute for Gender in Medicine, Center for Cardiovascular Research, Charité - Universitätsmedizin Berlin, Corporate Member of Freie Universität Berlin, Humboldt - Universität zu Berlin and Berlin Institute of Health, Berlin, Germany

[5]Department of Cardiology, University Hospital Zürich, University of Zürich, Zürich, Switzerland

[6]Center for Lifespan Changes in Brain and Cognition (LCBC), Dept of Psychology, University of Oslo, Oslo, Norway

[7]Charité - Universitätsmedizin Berlin, BCRT - Berlin Institute of Health Center for Regenerative Therapies, Berlin, Germany

Corresponding author:

Ilja Demuth (Ph.D.)
Charité - Universitätsmedizin Berlin
Lipid Clinic at the Interdisciplinary Metabolism Center,
Biology of Aging Group
Augustenburger Platz 1
13353 Berlin
Email: ilja.demuth@charite.de
Phone: ++49 30 450 569 143
FAX: ++49 30 450 566 904

Supplementary Table 1: Sex-stratified cohort characteristics of participants with diagnosed diabetes at baseline examination (n=126). Significance of differences between sexes was assessed by t-test and chi-squared test.

	Women					Men					
	n	Mean, %	SD	Min	Max	n	Mean, %	SD	Min	Max	p-value
Chronological Age (T0)	52	68.09	3.56	61.91	76.95	74	68.01	3.78	61.37	77.29	0.904
Chronological Age (T1)	74	75.26	4.27	66.78	85.94	52	75.74	3.83	68.91	85.61	0.520
7-CpG DNAmA (T0)	41	66.08	8.24	49.31	89.03	68	67.05	7.89	40.25	90.62	0.540
7-CpG DNAmAA (T0)	36	0.37	6.89	-16.01	13.90	65	0.95	7.24	-20.75	22.33	0.694
Smoking (packyears, T0)	39	7.72	13.47	0	45	62	20.24	20.08	0	80	0.001
Alcohol intake (g/d, T0)	40	12.10	14.98	0.62	65.16	68	17.90	17.30	0.44	86.40	0.081
BMI (T0)	43	29.60	4.54	21.04	39.87	67	29.27	3.91	19.41	39.62	0.684
Diabetes medication (yes, T0)	25	49.02				41	55.41				0.603
DCSI (T0)	52	0.71	1.26	0	7	74	1.42	1.42	0	5	0.005
DCSI (T1)	52	1.92	1.90	0	7	74	2.18	1.85	0	6	0.457
DCSI increase (yes)	28	53.85				41	55.41				1.000
Fasting Glucose (T0)	49	114.45	23.70	69.00	158.00	72	129.57	34.86	59.00	254.00	0.009
oGTT (T0)	20	175.75	62.21	94.00	308.00	22	209.91	57.36	94.00	307.00	0.071
HbA1c (T0)	48	6.40	0.70	5.00	8.70	71	6.54	0.74	5.20	8.90	0.299

Note: DNAmA = DNA methylation age, DNAmAA = DNA methylation age acceleration; T0 = baseline examination, T1 = follow-up examination, g/d = gram per day, BMI = body mass index, DCSI = Diabetes Complications Severity Index; oGTT = oral glucose tolerance test; HbA1c = glycated hemoglobin.

Supplementary Table 2: Logistic regression analyses of diagnosis of diabetes mellitus on DNAmAA of five different epigenetic clocks in cross-sectional data at follow-up. Model 1: no covariates; Model 2: chronological age; Model 3: Model 2 + sex (if applicable); Model 4: Model 3 + alcohol consumption (yes/no) + smoking (packyears) + antidiabetic medication (yes/no) + BMI. All variables were assessed at follow-up. All available participants are included in these analyses.

		Women And Men						Women					Men				
	Model	Estimate	SE	OR	p-value		n	Estimate	SE	OR	p-value	n	Estimate	SE	OR	p-value	n
7-CpG DNAmAA	1	0.00	0.01	1.00	0.959		1055	0.02	0.02	1.02	0.336	549	-0.03	0.02	0.97	0.096	506
	2	0.00	0.01	1.00	0.956		1055	0.02	0.02	1.02	0.302	549	-0.03	0.02	0.97	0.091	506
	3	-0.01	0.01	0.99	0.521		1055										
	4	-0.03	0.02	0.97	0.151		977	-0.01	0.03	0.99	0.801	515	-0.05	0.03	0.95	0.112	462
Horvath DNAmAA	1	0.01	0.02	1.01	0.516		1051	0.02	0.03	1.02	0.583	549	0.00	0.03	1.00	0.926	502
	2	0.01	0.02	1.01	0.513		1051	0.02	0.03	1.02	0.528	549	0.00	0.03	1.00	0.895	502
	3	0.01	0.02	1.01	0.768		1051										
	4	0.00	0.03	1.00	0.900		974	-0.01	0.05	0.99	0.762	515	0.01	0.04	1.01	0.752	459
Hannum DNAmAA	1	-0.01	0.02	0.99	0.720		1051	0.00	0.03	1.00	0.934	549	-0.04	0.03	0.96	0.216	502
	2	-0.01	0.02	0.99	0.721		1051	0.00	0.03	1.00	0.963	549	-0.04	0.03	0.96	0.209	502
	3	-0.02	0.02	0.98	0.310		1051										
	4	0.01	0.03	1.02	0.628		974	0.01	0.05	1.01	0.818	515	0.02	0.04	1.02	0.693	459
PhenoAge DNAmAA	1	0.03	0.01	1.03	0.038	*	1051	0.03	0.02	1.03	0.249	549	0.03	0.02	1.03	0.185	502
	2	0.03	0.01	1.03	0.037	*	1051	0.03	0.02	1.03	0.209	549	0.03	0.02	1.03	0.197	502
	3	0.03	0.02	1.03	0.076		1051										
	4	0.03	0.02	1.03	0.168		974	0.03	0.03	1.03	0.359	515	0.04	0.03	1.04	0.197	459
GrimAge DNAmAA	1	0.07	0.02	1.07	0.002	**	1051	0.03	0.04	1.03	0.538	549	0.06	0.03	1.06	0.066	502
	2	0.07	0.02	1.08	0.002	**	1051	0.03	0.04	1.03	0.486	549	0.06	0.03	1.06	0.071	502
	3	0.05	0.03	1.05	0.065		1051										
	4	0.06	0.04	1.06	0.148		974	0.09	0.06	1.09	0.156	515	0.02	0.06	1.03	0.659	459

Note: DNAmAA = DNA methylation age acceleration, SE = standard error, OR = odds ratio.

Supplementary Table 3: Logistic regression analyses of diagnosed T2D on 7-CpG DNAmAA in cross-sectional data at baseline. Model 1: unadjusted; Model 2: chronological age + sex (if applicable); Model 3: Model 2 + packyears + alcohol intake (g/day) + antidiabetic medication (yes/no) + BMI. Analyses were conducted with all available participants as well as with sex-stratified subgroups.

Model	Estimate	SE	OR	p-value	n
Women and Men					
1	0.020	0.016	1.021	0.189	921
2	0.012	0.016	1.012	0.449	921
3	0.010	0.024	1.010	0.669	834
Women					
1	0.040	0.026	1.041	0.126	458
2	0.040	0.026	1.041	0.127	458
3	0.013	0.037	1.013	0.733	419
Men					
1	-0.003	0.020	0.997	0.895	463
2	-0.004	0.020	0.996	0.835	463
3	0.010	0.033	1.010	0.766	415

Note: SE = standard error, OR = odds ratio

Supplementary Table 4: Logistic regression analyses of diagnosed T2D at follow-up on 7-CpG DNAmAA at baseline in longitudinal data of BASE-II participants. Model 1: unadjusted; Model 2: chronological age (years); Model 3: Model 2 + sex (if applicable); Model 4: Model 3 + alcohol intake (yes/no); Model 5: Model 4 + packyears; Model 6: Model 5 + BMI. Analyses were conducted with all available participants as well as with sex-stratified subgroups.

Model	Estimate	SE	OR	p-value	n
Women And Men					
1	0.00	0.01	1.00	0.822	920
2	0.00	0.01	1.00	0.823	920
3	0.00	0.01	1.00	0.732	920
4	0.00	0.01	1.00	0.736	920
5	-0.01	0.01	0.99	0.648	862
6	-0.01	0.01	0.99	0.624	861
Women					
1	0.02	0.02	1.02	0.337	458
3	0.02	0.02	1.02	0.307	458
4	0.02	0.02	1.02	0.307	458
5	0.03	0.02	1.03	0.233	436
6	0.03	0.02	1.03	0.250	436
Men					
1	-0.02	0.02	0.98	0.230	462
3	-0.02	0.02	0.98	0.218	462
4	-0.02	0.02	0.98	0.222	462
5	-0.03	0.02	0.97	0.119	426
6	-0.03	0.02	0.97	0.120	425

Note: SE = standard error, OR = odds ratio.

Supplementary Table 5: Linear regression analyses of blood parameters on DNAmAA of five epigenetic clocks in cross-sectional data at follow-up. Model 1: no covariates; Model 2: chronological age + sex + alcohol consumption (yes/no) + smoking (packyears) + antidiabetic medication (yes/no) + BMI. All variables were assessed at follow-up. All available participants were included in this analysis.

		Fasting Glucose					2h-oGTT					HbA1c			
	Model	Estimate	SE	p-value		n	Estimate	SE	p-value		n	Estimate	SE	p-value	n
7-CpG DNAmAA	1	0.16	0.11	0.135		1046	-0.03	0.20	0.901		823	0.00	0.00	0.498	1051
	2	0.04	0.09	0.677		969	0.11	0.21	0.596		765	0.00	0.00	0.934	973
Horvath DNAmAA	1	0.29	0.17	0.092		1042	0.07	0.32	0.833		820	0.00	0.00	0.724	1047
	2	0.19	0.14	0.161		966	0.35	0.33	0.300		762	0.00	0.00	0.915	970
Hannum DNAmAA	1	0.13	0.18	0.470		1042	0.58	0.33	0.080		820	-0.01	0.00	0.085	1047
	2	0.20	0.14	0.166		966	0.78	0.34	0.023	*	762	0.00	0.00	0.314	970
PhenoAge DNAmAA	1	0.44	0.13	0.001	***	1042	0.68	0.24	0.005	**	820	0.01	0.00	0.071	1047
	2	0.26	0.10	0.013	*	966	0.75	0.26	0.003	**	762	0.00	0.00	0.201	970
GrimAge DNAmAA	1	0.81	0.20	<0.001	***	1042	-0.06	0.38	0.877		820	0.01	0.00	0.055	1047
	2	0.25	0.19	0.183		966	0.01	0.46	0.980		762	0.00	0.00	0.441	970

Note: DNAmAA = DNA methylation age acceleration, SE = standard error, HbA1c = glycated hemoglobin, oGTT = oral glucose tolerance test.

Supplementary Table 6: Sex-stratified linear regression analyses of blood parameters on DNAmAA of five epigenetic clocks in cross-sectional data at follow-up. Model 1: no covariates; Model 2: chronological age + alcohol consumption (yes/no) + smoking (packyears) + antidiabetic medication (yes/no) + BMI. All variables were assessed at follow-up.

		Fasting Glucose					oGTT					HbA1c			
Women	Model	Estimate	SE	p-value		n	Estimate	SE	p-value		n	Estimate	SE	p-value	n
7-CpG DNAmAA	1	0.14	0.13	0.299		542	-0.13	0.27	0.624		435	0.00	0.00	0.496	546
	2	-0.04	0.10	0.700		509	0.04	0.27	0.894		409	0.00	0.00	0.419	512
Horvath DNAmAA	1	0.13	0.21	0.530		542	-0.40	0.43	0.354		435	0.00	0.01	0.713	546
	2	0.00	0.16	0.984		509	-0.23	0.45	0.603		409	-0.01	0.00	0.222	512
Hannum DNAmAA	1	0.01	0.23	0.962		542	0.46	0.46	0.325		435	-0.01	0.01	0.101	546
	2	0.11	0.17	0.525		509	0.57	0.46	0.215		409	-0.01	0.00	0.087	512
PhenoAge DNAmAA	1	0.31	0.15	0.042	*	542	0.49	0.32	0.130		435	0.00	0.00	0.416	546
	2	0.21	0.12	0.081		509	0.57	0.34	0.099		409	0.00	0.00	0.538	512
GrimAge DNAmAA	1	0.29	0.28	0.307		542	-0.04	0.57	0.942		435	0.00	0.01	0.999	546
	2	0.32	0.22	0.148		509	0.11	0.61	0.862		409	0.00	0.01	0.562	512
Men															
7-CpG DNAmAA	1	0.00	0.17	0.990		504	0.08	0.32	0.800		388	0.00	0.00	0.878	505
	2	0.15	0.15	0.292		460	0.21	0.34	0.527		356	0.00	0.00	0.532	461
Horvath DNAmAA	1	0.27	0.27	0.334		500	0.56	0.48	0.244		385	0.00	0.01	0.626	501
	2	0.43	0.22	0.049	*	457	0.95	0.50	0.059		353	0.01	0.01	0.219	458
Hannum DNAmAA	1	-0.10	0.28	0.711		500	0.73	0.49	0.139		385	-0.01	0.01	0.153	501
	2	0.30	0.23	0.191		457	0.99	0.52	0.057		353	0.00	0.01	0.978	458
PhenoAge DNAmAA	1	0.45	0.21	0.031	*	500	0.92	0.37	0.013	*	385	0.01	0.00	0.154	501
	2	0.33	0.17	0.052		457	0.96	0.39	0.014	*	353	0.00	0.00	0.249	458
GrimAge DNAmAA	1	0.61	0.34	0.073		500	-0.13	0.60	0.827		385	0.01	0.01	0.132	501
	2	0.19	0.30	0.529		457	-0.11	0.69	0.873		353	0.00	0.01	0.592	458

Note: DNAmAA = DNA methylation age acceleration, SE = standard error, HbA1c = glycated hemoglobin, oGTT = oral glucose tolerance test.

Supplementary Table 7: Linear regression analyses of DCSI on DNAmAA and covariates in cross-sectional data at baseline. Included are longitudinally available participants with diagnosed diabetes mellitus at baseline. Model 1: no covariates; Model 2: chronological age + sex (if applicable); Model 3: Model 2 + alcohol consumption (yes/no) + smoking (packyears) + antidiabetic medication (yes/no) + BMI. All variables were assessed at baseline.

Model	Estimate	SE	p-value	n
Women and Men				
1	0.03	0.02	0.138	126
2	0.01	0.02	0.717	126
3	0.01	0.02	0.626	96
Men				
1	0.03	0.02	0.175	74
2	0.01	0.02	0.678	74
3	0.02	0.03	0.399	59
Women				
1	0.01	0.02	0.739	52
2	0.00	0.02	0.999	52
3	-0.02	0.03	0.587	37

Note: SE = standard error.

Supplementary Table 8: Descriptive statistics of all available participants at follow-up examination. Significance of differences between sexes was assessed by t-test or chi-squared test. In addition to the 1,083 BASE II participants assessed at baseline and follow-up, 17 participants were assessed only during the medical part of the follow-up examination.

	Women and Men					Women					Men					
	n	Mean, %	SD	Min	Max	n	Mean, %	SD	Min	Max	n	Mean, %	SD	Min	Max	p-value
Chronological age (T1)	1100	75.60	3.77	64.91	94.07	573	75.72	3.53	66.41	94.07	527	75.48	4.01	64.91	90.03	0.276
Sex																
male	527	47.91									527	100.00				
female	573	52.09				573	100.00									
Smoking (packyears, T1)	1019	9.79	17.61	0.00	150.00	537	6.30	13.48	0.00	114.00	482	13.68	20.61	0.00	150.00	<0.001
Alcohol consumption (T1)																
no	185	16.86				106	18.53				79	15.05				0.145
yes	912	83.14				466	81.47				446	84.95				
BMI (T1)	1098	26.97	4.25	17.17	49.68	573	26.63	4.68	17.17	49.68	525	27.35	3.69	20.02	41.77	0.005
7-CpG DNAmAA (T1)	1071	0.03	6.42	-24.93	34.48	558	-1.02	6.35	-24.37	25.30	513	1.17	6.31	-24.93	34.48	<0.001
Horvath DNAmAA (T1)	1067	0.03	4.04	-12.31	23.45	558	-0.43	3.98	-12.31	23.45	509	0.54	4.04	-8.94	17.44	<0.001
Hannum DNAmAA (T1)	1067	0.01	3.89	-10.80	28.57	558	-0.72	3.68	-10.80	12.73	509	0.81	3.96	-9.32	28.57	<0.001
PhenoAge DNAmAA (T1)	1067	0.04	5.42	-16.54	25.80	558	-0.48	5.39	-16.54	25.80	509	0.62	5.39	-13.51	20.94	0.001
GrimAge DNAmAA (T1)	1067	0.03	3.39	-10.82	12.84	558	-1.30	2.93	-10.82	10.71	509	1.47	3.27	-8.17	12.84	<0.001
Fasting glucose (T1)	1070	102.22	22.26	65.00	304.00	554	98.77	19.12	65.00	231.00	516	105.92	24.69	72.00	304.00	<0.001
oGTT (T1)	837	118.46	37.39	32.00	358.00	442	117.94	35.59	44.00	244.00	395	119.04	39.34	32.00	358.00	0.671
HbA1c (T1)	1072	5.73	0.54	4.40	10.00	555	5.70	0.50	4.60	9.70	517	5.76	0.59	4.40	10.00	0.052
DCSI (T1)	1083	1.26	1.49	0.00	8.00	563	1.21	1.41	0.00	8.00	520	1.33	1.56	0.00	7.00	0.181
Antidiabetic medication (T1)																
no	983	90.77				528	93.78				455	87.50				0.001
yes	100	9.23				35	6.22				65	12.50				
Diagnosed diabetes mellitus (T1)																
no	896	82.89				487	86.50				409	78.96				0.001
yes	185	17.11				76	13.50				109	21.04				

Note: DNAmA = DNA methylation age, DNAmAA = DNA methylation age acceleration, T0 = baseline examination, T1 = follow-up examination, BMI = body mass index, DCSI = Diabetes Complications Severity Index, oGTT = oral glucose tolerance test, HbA1c = glycated hemoglobin.

Supplementary Table 9: Linear regression analyses of DCSI on DNAmAA and covariates in cross-sectional data at follow-up. Included are participants with diagnosed diabetes mellitus at follow-up. Model 1: no covariates; Model 2: chronological age, sex (if applicable); Model 3: Model 2 + alcohol consumption (yes/no) + smoking (packyears) + antidiabetic medication (yes/no) + BMI. All variables were assessed at follow-up.

		Women and Men					Women				Men				
	Model	Estimate	SE	p-value		n	Estimate	SE	p-value	n	Estimate	SE	p-value		n
7-CpG DNAmAA	1	0.05	0.02	0.029	*	180	0.03	0.03	0.387	73	0.06	0.03	0.040	*	107
	2	0.05	0.02	0.012	*	180	0.04	0.03	0.285	73	0.07	0.03	0.018	*	107
	3	0.04	0.02	0.087		163	0.03	0.03	0.372	67	0.05	0.03	0.114		96
Horvath DNAmAA	1	-0.02	0.03	0.525		179	-0.05	0.06	0.408	73	-0.01	0.04	0.800		106
	2	-0.01	0.03	0.680		179	-0.04	0.06	0.525	73	0.00	0.04	0.957		106
	3	-0.03	0.04	0.377		163	-0.04	0.06	0.488	67	-0.01	0.04	0.787		96
Hannum DNAmAA	1	0.02	0.03	0.491		179	0.06	0.06	0.343	73	0.00	0.04	0.915		106
	2	0.01	0.03	0.742		179	0.05	0.06	0.379	73	-0.01	0.04	0.842		106
	3	0.00	0.03	0.974		163	0.07	0.06	0.264	67	-0.03	0.04	0.479		96
PhenoAge DNAmAA	1	0.03	0.02	0.242		179	0.00	0.04	0.982	73	0.05	0.03	0.133		106
	2	0.04	0.02	0.153		179	0.01	0.04	0.795	73	0.05	0.03	0.098		106
	3	0.03	0.03	0.302		163	0.00	0.04	0.945	67	0.02	0.03	0.474		96
GrimAge DNAmAA	1	-0.02	0.04	0.638		179	-0.10	0.08	0.222	73	-0.01	0.05	0.816		106
	2	-0.03	0.04	0.477		179	-0.08	0.08	0.356	73	-0.01	0.05	0.769		106
	3	-0.06	0.05	0.208		163	-0.14	0.09	0.152	67	-0.04	0.06	0.473		96

Note: DNAmAA = DNA methylation age acceleration, SE = standard error.

Supplementary Table 10: Logistic regression of increase in DCSI of one or more points vs. no increase in DCSI (dichotomized) on 7-CpG DNAmAA at baseline. Model 1: no covariates; Model 2: DCSI (T0); Model 3: Model 2 + chronological age (T0) + sex (if applicable); Model 4: Model 3 + smoking (packyears, T0) + alcohol consumption (g/d, T0) + diabetes medication (yes/no, T0) + BMI (T0). Included are all participants with diagnosed diabetes mellitus at baseline.

Model	Estimate	SE	OR	p-value		n
Women and Men						
1	0.047	0.030	1.048	0.114		101
2	0.046	0.030	1.047	0.127		101
3	0.048	0.030	1.049	0.117		101
4	0.040	0.033	1.041	0.219		89
Women						
1	-0.040	0.051	0.961	0.429		36
2	-0.042	0.051	0.959	0.414		36
3	-0.037	0.052	0.964	0.477		36
4	-0.004	0.061	0.996	0.949		33
Men						
1	0.100	0.042	1.105	0.017	*	65
2	0.099	0.042	1.104	0.019	*	65
3	0.100	0.043	1.105	0.019	*	65
4	0.101	0.051	1.106	0.045	*	56

Note: T0 = baseline, SE = standard error, OR = odds ratio.

Supplementary Table 11: Linear regression analysis of change in DCSI per year of follow-up time on 7-CpG DNAmAA at baseline. Model 1: no covariates; Model 2: chronological age (years, T0); Model 3: Model 2 + sex (if applicable); Model 4: Model 3 + smoking (packyears, T0), Model 5: Model 4 + alcohol intake (g/day, T0); Model 6: Model 5 + antidiabetic medication; Model 7: Model 6 + BMI (T0).

Model		Estimate	SE	p-value		n
Women and Men						
	1	0.006	0.003	0.080		101
	2	0.006	0.003	0.082		101
	3	0.006	0.003	0.076		101
	4	0.006	0.003	0.086		94
	5	0.006	0.003	0.094		93
	6	0.006	0.003	0.090		93
	7	0.005	0.003	0.189		89
Women						
	1	-0.005	0.005	0.376		36
	2	-0.004	0.005	0.479		36
	4	-0.003	0.005	0.590		35
	5	-0.003	0.005	0.589		34
	6	-0.002	0.006	0.745		34
	7	-0.002	0.006	0.772		33
Men						
	1	0.011	0.004	0.007	**	65
	2	0.011	0.004	0.008	**	65
	4	0.011	0.004	0.012	*	59
	5	0.010	0.004	0.013	*	59
	6	0.010	0.004	0.014	*	59
	7	0.009	0.004	0.042	*	56

Note: SE = standard error.

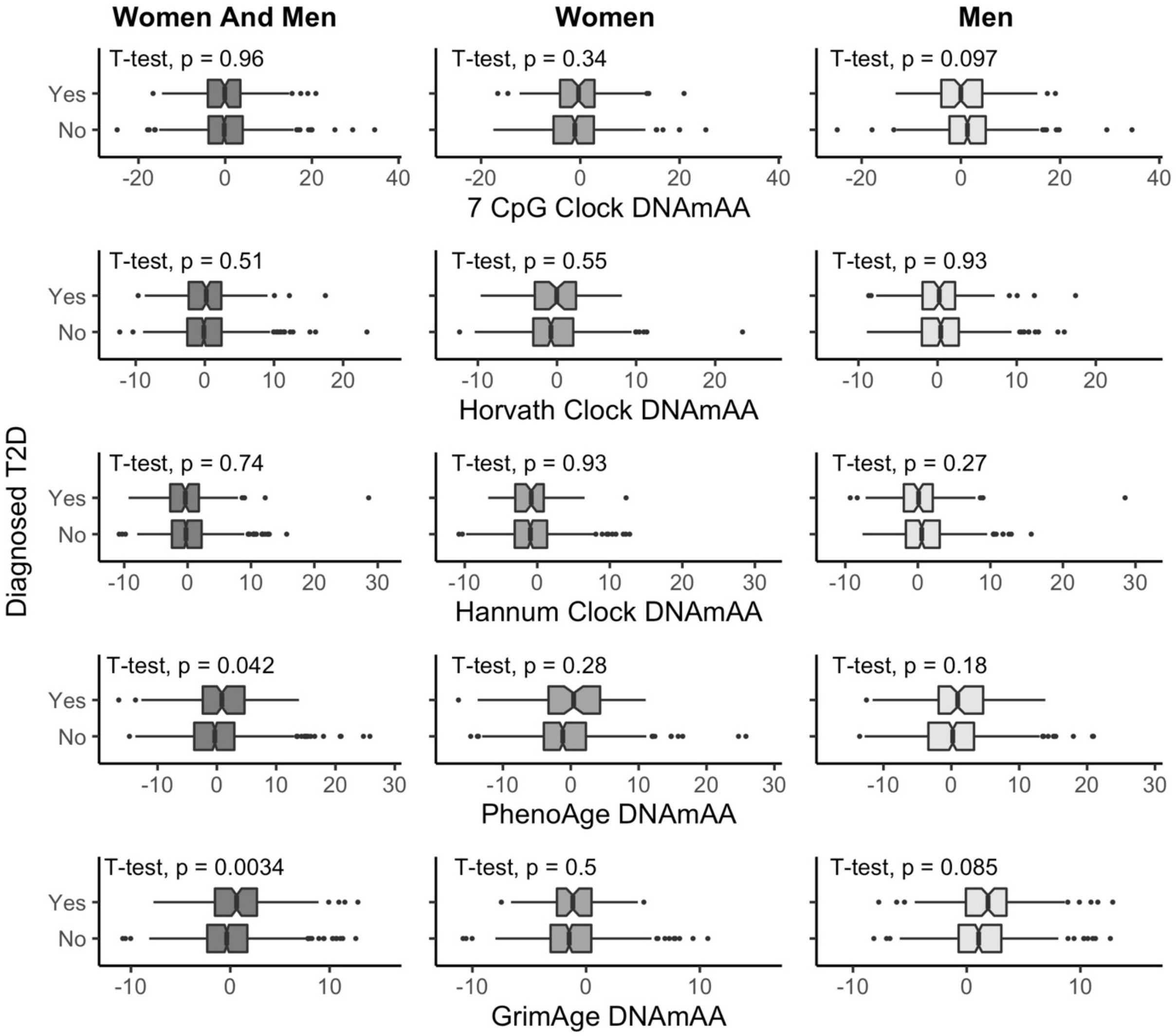

Supplementary Figure 1: Boxplots of DNAmAA estimates by five different epigenetic clocks in the whole dataset as well as the female and male subgroup, stratified by their diagnostic status (T2D vs. no T2D). Median, hinges (25th and 75th percentile) as well as Tukey-style whiskers (1.5* inter-quartile-range) are displayed. The notch approximates the 95% confidence interval around the median.
