# Supplementary material for: DNA methylation age acceleration is associated with risk of diabetes complications
Source: Commun Med (Lond). 2023 Feb 10;3:21. doi: 10.1038/s43856-023-00250-8 (PMC9918553; doi:10.1038/s43856-023-00250-8)
Supplement: Supplementary file 3 — Description of Additional Supplementary Files [file 43856_2023_250_MOESM3_ESM.pdf]

Description of Additional Supplementary Files

File Name: Supplementary Data

Description: Source data for figure 3
