# Supplementary material for: DNA methylation age acceleration is associated with risk of diabetes complications
Source: Commun Med (Lond). 2023 Feb 10;3:21. doi: 10.1038/s43856-023-00250-8 (PMC9918553; doi:10.1038/s43856-023-00250-8)
Supplement: Supplementary file 4 — Reporting Summary [file 43856_2023_250_MOESM4_ESM.pdf]

nature research

Corresponding author(s): Ilja Demuth

Last updated by author(s): Nov 25, 2022

Reporting Summary

Nature Research wishes to improve the reproducibility of the work that we publish. This form provides structure for consistency and transparency in reporting. For further information on Nature Research policies, see our Editorial Policies and the Editorial Policy Checklist.

Statistics

For all statistical analyses, confirm that the following items are present in the figure legend, table legend, main text, or Methods section.

n/a	Confirmed	
☐	☒	The exact sample size (n) for each experimental group/condition, given as a discrete number and unit of measurement
☐	☒	A statement on whether measurements were taken from distinct samples or whether the same sample was measured repeatedly
☐	☒	The statistical test(s) used AND whether they are one- or two-sided<br>*Only common tests should be described solely by name; describe more complex techniques in the Methods section.*
☐	☒	A description of all covariates tested
☐	☒	A description of any assumptions or corrections, such as tests of normality and adjustment for multiple comparisons
☐	☒	A full description of the statistical parameters including central tendency (e.g. means) or other basic estimates (e.g. regression coefficient) AND variation (e.g. standard deviation) or associated estimates of uncertainty (e.g. confidence intervals)
☐	☒	For null hypothesis testing, the test statistic (e.g. F, t, r) with confidence intervals, effect sizes, degrees of freedom and P value noted<br>*Give P values as exact values whenever suitable.*
☒	☐	For Bayesian analysis, information on the choice of priors and Markov chain Monte Carlo settings
☒	☐	For hierarchical and complex designs, identification of the appropriate level for tests and full reporting of outcomes
☐	☒	Estimates of effect sizes (e.g. Cohen's d, Pearson's r), indicating how they were calculated

Our web collection on statistics for biologists contains articles on many of the points above.

Software and code

Policy information about availability of computer code

Data collection	No computer code was necessary for data collection.
Data analysis	Statistical analyses and all figures were conducted in R 3.6.2. ROC curves were calculated with the proc package. Significance of difference between ROC curves was computed with the “roc.test” function that employs the approach described by DeLong and colleagues.

For manuscripts utilizing custom algorithms or software that are central to the research but not yet described in published literature, software must be made available to editors and reviewers. We strongly encourage code deposition in a community repository (e.g. GitHub). See the Nature Research guidelines for submitting code & software for further information.

Data

Policy information about availability of data

All manuscripts must include a data availability statement. This statement should provide the following information, where applicable:

- Accession codes, unique identifiers, or web links for publicly available datasets
- A list of figures that have associated raw data
- A description of any restrictions on data availability

Source data for figure 3 is provided as Supplementary Data. Due to concerns for participants privacy as well as data protection regulations, BASE-II raw data cannot be made publicly available. Because of their solely descriptive nature, this applies to source data for figures 1 and 2 as well. Interested investigators are invited to contact the scientific coordinator of BASE-II, Ludmila Müller (lmueller@mpib-berlin.mpg.de), to obtain source data for figures 1 and 2 or apply for raw data access. Additional information can be found on the BASE-II website: https://www.base2.mpg.de/7549/data-documentation.

Field-specific reporting

Please select the one below that is the best fit for your research. If you are not sure, read the appropriate sections before making your selection.

☒ Life sciences ☐ Behavioural & social sciences ☐ Ecological, evolutionary & environmental sciences

For a reference copy of the document with all sections, see nature.com/documents/nr-reporting-summary-flat.pdf

Life sciences study design

All studies must disclose on these points even when the disclosure is negative.

Sample size	The medical examination of the Berlin Aging Study II (BASE-II) included 1,671 participants between the age of 60-85 years as assessed at baseline between 2009 and 2014. An additional group of 500 younger participants was assessed as well (age range 20 to 37 years) but is not analyzed in this study. After an average follow-up period of 7.4 years, 1,083 participants of the older age group were reassessed as part of the GendAge study and are therefore available for longitudinal analyses. An additional 17 participants were only assessed at follow-up examination.
Data exclusions	No data was systematically excluded, however, an available case analysis was performed. Therefore, participants who did not provide information on all variables needed for an analysis were excluded from it. The analyzed sample size is indicated for each analysis.
Replication	No replications on independent samples were done for this study.
Randomization	The study design did not require randomization (no intervention).
Blinding	The study design did not require blinding (no intervention).

Reporting for specific materials, systems and methods

We require information from authors about some types of materials, experimental systems and methods used in many studies. Here, indicate whether each material, system or method listed is relevant to your study. If you are not sure if a list item applies to your research, read the appropriate section before selecting a response.

Materials & experimental systems

n/a	Involved in the study	
☒	☐	Antibodies
☒	☐	Eukaryotic cell lines
☒	☐	Palaeontology and archaeology
☒	☐	Animals and other organisms
☐	☒	Human research participants
☒	☐	Clinical data
☒	☐	Dual use research of concern

Methods

n/a	Involved in the study	
☒	☐	ChIP-seq
☒	☐	Flow cytometry
☒	☐	MRI-based neuroimaging

Human research participants

Policy information about studies involving human research participants

Population characteristics	The BASE-II is an explorative multi-disciplinary study that examines factors promoting "healthy" vs. "unhealthy" aging. The medical part of this study included 1,671 participants of the greater Berlin metropolitan area between the age of 60-85 years as assessed at baseline between 2009 and 2014. An additional group of 500 younger participants was assessed as well (age range 20 to 37 years) but is not analyzed in this study. After an average follow-up period of 7.4 years, 1,083 participants of the older age group were reassessed as part of the GendAge study and are therefore available for longitudinal analyses. An additional 17 participants were only assessed at follow-up examination. The mean age of BASE-II participants with diagnosed T2D at baseline and a completed follow-up examination was 68.0 years (SD= 3.7 years, n=126, 41.3 % female) at baseline and 75.5 years (SD= 4.1 years) at follow-up.
Recruitment	Recruitment was based on information displayed in the public transport system and local newspapers.
Ethics oversight	The Ethics Committee of the Charité – Universitätsmedizin Berlin approved the studies (approval numbers EA2/029/09 and EA2/144/16).

Note that full information on the approval of the study protocol must also be provided in the manuscript.
